# A Novel Rule-Based Approach in Mapping Landslide Susceptibility

**DOI:** 10.3390/s19102274

**Published:** 2019-05-16

**Authors:** Majid Shadman Roodposhti, Jagannath Aryal, Biswajeet Pradhan

**Affiliations:** 1 Discipline of Geography and Spatial Sciences, School of Technology, Environments and Design, University of Tasmania, Churchill Ave, Hobart, TAS 7005, Australia; jagannath.aryal@utas.edu.au; 2 Centre for Advanced Modelling and Geospatial Information Systems (CAMGIS), University of Technology Sydney, NSW 2007, Australia; Biswajeet.Pradhan@uts.edu.au; 3 Department of Energy and Mineral Resources Engineering, Choongmu-gwan, Sejong University, 209 Neungdongro Gwangjin-gu, Seoul 05006, Korea

**Keywords:** Shannon entropy, uncertainty, landslide susceptibility mapping (LSM), GIS, Tasmania

## Abstract

Despite recent advances in developing landslide susceptibility mapping (LSM) techniques, resultant maps are often not transparent, and susceptibility rules are barely made explicit. This weakens the proper understanding of conditioning criteria involved in shaping landslide events at the local scale. Further, a high level of subjectivity in re-classifying susceptibility scores into various classes often downgrades the quality of those maps. Here, we apply a novel rule-based system as an alternative approach for LSM. Therein, the initially assembled rules relate landslide-conditioning factors within individual rule-sets. This is implemented without the complication of applying logical or relational operators. To achieve this, first, Shannon entropy was employed to assess the priority order of landslide-conditioning factors and the uncertainty of each rule within the corresponding rule-sets. Next, the rule-level uncertainties were mapped and used to asses the reliability of the susceptibility map at the local scale (i.e., at pixel-level). A set of If-Then rules were applied to convert susceptibility values to susceptibility classes, where less level of subjectivity is guaranteed. In a case study of Northwest Tasmania in Australia, the performance of the proposed method was assessed by receiver operating characteristics’ area under the curve (AUC). Our method demonstrated promising performance with AUC of 0.934. This was a result of a transparent rule-based approach, where priorities and state/value of landslide-conditioning factors for each pixel were identified. In addition, the uncertainty of susceptibility rules can be readily accessed, interpreted, and replicated. The achieved results demonstrate that the proposed rule-based method is beneficial to derive insights into LSM processes.

## 1. Introduction

There are a number of natural hazards, which are a threat to both human lives and properties throughout the world. These include flooding, bushfire, dust storms, coastal processes, and landslides. Many spatial scientists have contributed to prevent or reduce actual damage from natural hazards through modeling and production of susceptibility maps [1,2,3,4,5,6,7,8,9]. Obviously, susceptibility mapping can be a crucial tool for a wide range of end-users, from both private and public sectors, aimed at hazard mitigation purposes at both local and international levels. Although susceptibility maps may not be the best approach for confronting all existing types of natural hazards, in terms of landslide events, they are both popular and effective [10]. 

In terms of landslides, up to now, various methodological approaches including heuristic [11,12,13], deterministic [14,15] and statistical [6,16,17] methods have been applied for landslide susceptibility mapping (LSM). While heuristic methods are subjective to the experts’ knowledge, deterministic approaches restricted to small-scale areas (i.e., 1:25,000–1:5,000) [8,18,19,20]. This is because of the fact that in deterministic models there is a need for detailed information on lithological units, soil characteristics, slope geometry, discontinuity characteristics, and hydrological conditions of the slopes. Therefore, considering the limitations that apply to heuristic and deterministic methods, statistical approaches have been more popular for the implementation of susceptibility maps [10].

Taking natural hazards into account, a susceptibility map identifies areas, which are more or less prone to a potential hazard occurrence using low to high possibility values/classes [3,10]. However, for effective mitigation of natural hazards using management plans, the conditioning factors of a potential hazard must be also highlighted throughout the spatial domain [10,21]. LSM depicts where the slope failure may occur spatially [2,10,12,21]. Although to date, statistical methodologies developed for quantifying variations of landslide susceptibility throughout spatial domain are usually accurate, the latter requirement of highlighting conditioning factors in a transparent way is often neglected, especially at the pixel-level [21]. This simply means that it is not often possible for the experts to retrieve the classification rules at pixel-level. Nonetheless, the proper understanding of the conditioning factors and scientific methodologies is also crucial to our understanding of mechanisms of landslides at local (i.e., pixel) level [22,23]. 

Another disadvantage that limits the application of susceptibility assessment is the subjective re-classification of susceptibility scores into susceptibility classes (i.e., high, medium and low susceptibility) using quantile [24], equal interval [13], natural breaks [11,25] or other classification schemes. This is intertwined with the drawback of subjectivity that is the main limitation of heuristic methods [26], which can also downgrade “*statistical methods*”. This is usually because of the fact that there is a risk that experts/modelers’ expectations will affect the final interpretation and the experts/modelers will see what they want to see. 

Up to now, many researchers applied different approaches that were aimed at developing novel LSM algorithms, while few may have considered to simultaneously tackle above-mentioned issues. Considering each one of the above issues individually, a smaller number of researchers considered highlighting landslide susceptibility rules at the pixel-level compared with reducing subjectivity. For instance, Chen, et al. [27] applied a new ensemble model by combining ANN, MaxEnt, and SVM machine-learning algorithms for LSM purposes. However, no susceptibility rule can be retrieved at the pixel-level using this approach. This limitation applies to a broad range of statistical and machine-learning approaches that are highly popular in contemporary LSM literature [28]. However, in terms of the latter issue, many researchers attempted to assess and/or resolve the subjectivity issue of LSM algorithms individually. For instance, Althuwaynee, et al. [29] assessed the efficiency of LSM models while reducing the subjectivity of knowledge-based approaches. Yan, et al. [30] proposed a novel hybrid approach for LSM by integrating analytical hierarchy process and normalized frequency ratio methods with the cloud model. A major contribution of this method was applying the cloud model to reduce the high level of subjectivity induced by experts’ opinions in the analytical hierarchy process method.

To account for above-mentioned problems, we apply and further modify a rule-based algorithm, DoTRules—Dictionary of Trusted Rules—for LSM that was originally applied for transition potential mapping in cellular automata land-use change models [31]. Our novel algorithm features identification of transparent rules that define the impact of key conditioning landslide factors at a pixel-level. Here every unique rule corresponds with at least one pixel of the susceptibility map, as well as an uncertainty value which quantifies the reliability of the same rule. In the proposed method, Shannon entropy is benefited to calculate the uncertainty of each susceptibility rule. Exploring susceptibility rules and their equivalent uncertainties is a beneficial provision to better understand the core rules involved in slope failure dynamics, which is useful for the implementation of hazard mitigation plans. In addition, following the recent attempts to reduce the level of subjectivity that exists in re-classification of susceptibility scores into susceptibility classes [32], we applied probability values of classification rules for the final implementation of susceptibility map using a set of “If-Then” rules. Although various forms of subjectivity may exist in the process of LSM, in the current research, we only focus on the conversion of susceptibility scores into susceptibility classes.

This study contributes to the better understanding of landslide mechanism at the local level by introducing a novel transparent rule-based algorithm, where no logical or relational operator is used. In addition, the proposed algorithm reduces the level of subjectivity that exists in the re-classification of susceptibility scores into susceptibility classes. As the applications of susceptibility maps are not only limited to landslide events, these are both valuable assets for better designing of hazard mitigation plans that are developed based on susceptibility maps. Here, we describe the modified version of DoTRules algorithm for LSM and demonstrate its application to Northwest Tasmania, Australia. We discuss the pros and cons of the new LSM approach more generally from the preparation of landslide susceptibility maps to broader environmental processes where it is necessary to understand the dynamic mechanism of conditioning factors involved in shaping natural hazards susceptibility.

## 2. Description of the Study Region

Landslides, for the most part of Australia, are not seen as an utmost threat to urban communities. However, this general belief is far from the reality of the situation where the history of landslides is highlighted with devastating events [33]. Since 1842, a total of 88 people have lost their lives due to 38 fatal landslides [34]. It is almost certain that these statistics are incomplete and that the real number of fatalities is much higher. 

**Figure 1:**
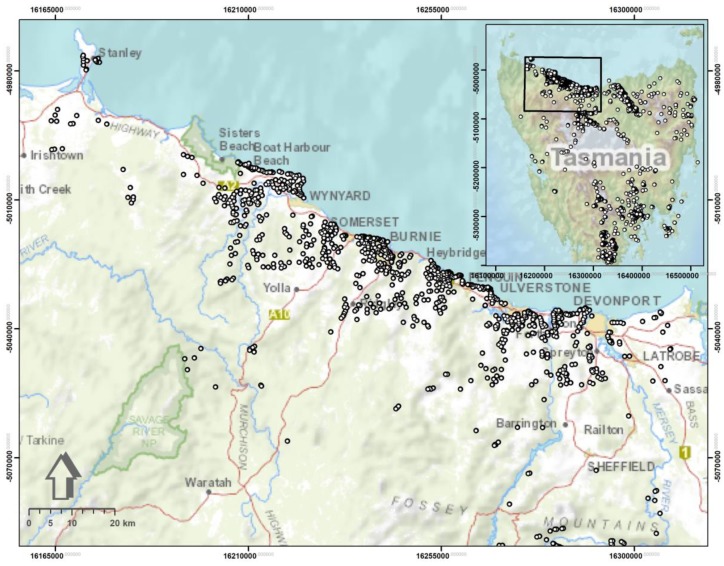
A location map of the study area. Dots represent the location of occurred various landslide events.

In terms of Tasmania, numerous patches of land are susceptible to slope instability, whereby the existing records since the 1950s, around 75 houses are seriously damaged by landslides, or demolished due to substantial damage. Fortunately, there is no occurred fatal incident during this time window. Nonetheless, these slope failure events are extremely disturbing to those directly affected, where the financial damage to people and/or the State runs into many millions of dollars. Yet, usually no insurance company provides cover for landslide damages, nor the Tasmanian government has paid to compensate for financial loss in the past [35]. No doubt that this type of disasters and its adverse effects can be avoided when ground conditions are deciphered and fully understood prior to the construction of properties. Although landslide events can occur anywhere across Tasmania, they have been mostly active in several areas of the Northwest landscape, the Tamar Valley, as well as specific areas in and around Hobart, Launceston, and St Helens. Here, in this research, we utilize a landslide inventory of Northwest Tasmania (Figure 1). Mineral Resources Tasmania (MRT) undertook many activities to deal with landslides such as LSM, administration of declared or proclaimed areas of slope failure and also monitoring of a small number of “*problematic*” landslides. The current landslide inventory that is utilized for this research is also developed by MRT (https://maps.thelist.tas.gov.au/listmap/app/list/map).

The high frequency and variation of landslide events in the area is due to a range of factors such as varied topography, climates (including rainfall patterns), and especially a combination of geologic and geomorphic (land forming) processes. In Tasmania, some slides (i.e., shallow slides) are often formed in soils developed on Tertiary basalt, sediments and colluvial material. Some others (i.e., deep-seated landslides) are developed where the unstable slope spreads well below shallow soils into deeply weathered regolith and/or underlying geological units [36,37]. There are 3030 slope instability records in the entire state of Tasmania, out of which the selected study area in the Northwest contains 1152-recorded events. Five major types of landslide movement including slide, flow, fall, topple, and spread are seen in Tasmania as well as in the studied area, where flows have been the most common types up to now. In many cases, landslides are actually a combination of different movement types. Among all the recorded events of Northwest Tasmania, 641 points belong to slides category (both shallow and deep-seated) are considered in this study. These are a down-slope movement of material along a distinctive surface of weakness such as a fault, joint, or bedding plane and can occur on slopes within the minimum and maximum range of 6°–14°, respectively. No doubt, this entangles the process of LSM [38].

## 3. Materials and Methods

### 3.1. Landslide-conditioning Factors 

**Figure 2:**
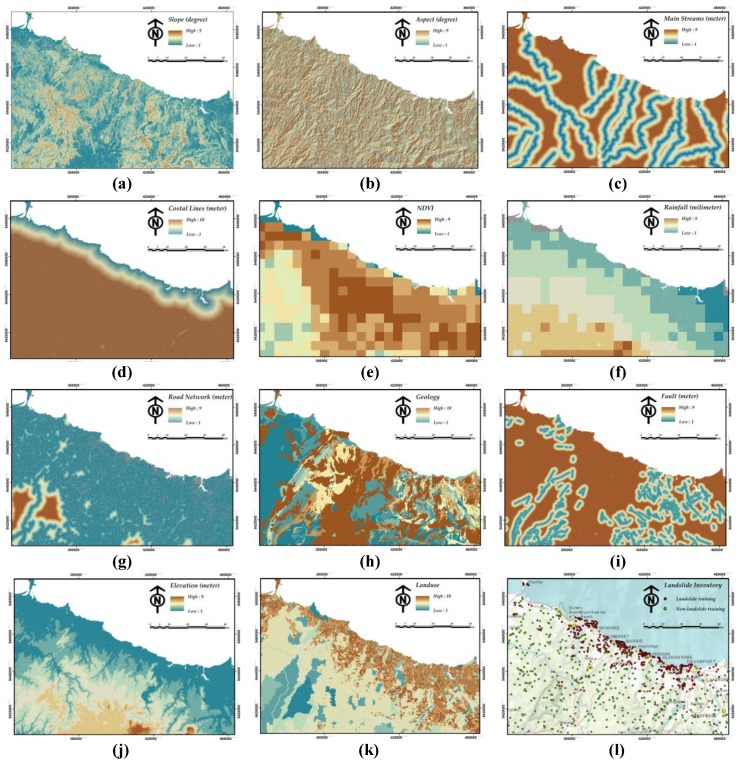
Eleven applied landslide-conditioning factors involving: (**a**) Slope; (**b**) aspect; (**c**) distance to main streams; (**d**) distance to coastal lines; (**e**) NDVI; (**f**) mean annual rainfall; (**g**) distance to roads; (**h**) geology; (**i**) distance to faults; (**j**) elevation; (**k**) land-use, and (**l**) landslide/non-landslide inventory database used for training the model.

In the process of selecting landslide conditioning factor, an initial study of local landslide properties and their spatial distribution was considered using available MRT reports. Afterward, with respect to the available peer-reviewed GIS-based LSM research [39,40,41], an initial group of landslide-conditioning factors of the study area as well as an inventory of 641 occurred landslide events, have been selected. Then we assessed the correlations among landslide occurrence and ten conditioning factors [42] including slope, aspect, distance to main streams, distance to coastal lines, NDVI, mean annual rainfall, distance to roads, geology, distance to faults, elevation and land-use for Northwest Tasmania. Based on the findings, we selected the ten aforementioned factors for landslide modeling for this study (Table 1 and Figure 2). In terms of NDVI data, it was ideal to apply a time series that was overlapping with rainfall data; however, as we did not have access to such data, a mean NDVI raster of Jun 2017–Jun 2018 was applied. This was the most recent data during the methodology implementation.

A typical grid cell approach was applied to assess landslide susceptibility because it is the most popular method referring to the current literature [43]. Here, all 11 landslide-conditioning factors were at different resolution; therefore, they were spatially resampled to 10 × 10 meter cell resolution, where the whole study area is composed of 11782 × 8303 cells. 

As per the model requirement, all landslide conditioning factors (Table 1) must be re-classified into H discrete classes. This can be simply done by re-classifying data with reference to existing literature or analyzing training data. Clustering is a second option for this purpose which is a more objective approach. In this paper, we have re-classified the data and selected these intervals by referring to the existing LSM literature. In terms of landslide points, almost all of the selected landslides belong to slide-type landslides. In terms of landslide inventory, 250 landslides were randomly selected for training purpose while 391 landslides were selected to test the susceptibility map. The preparation of data used for this research was done in ArcGIS 10.4 environment. 

A 10-meter digital elevation model (DEM) form Mineral Resources Tasmania (MRT) was used to model topographical features including elevation, slope, and aspect, where each of these layers was re-classified into nine different classes. Topographical data are conventionally applied in LSM models [44,45]. According to the training data, the landslides points are highly frequent in low elevation class (i.e., class 1) whereas they are almost normally distributed throughout different classes of the slope with a maximum frequency at class 3 (≈7°–12°). In the case of aspect, a bimodal trend can be observed, wherein landslide points are highly frequent around class 1 and 9 (i.e., Northwest and Northeast).

In this study, four distance layers were used namely, distance from main streams [46], coastal lines [47], roads [47], and faults [48]. They were prepared using the Euclidean distance tool in ArcGIS environment. The first two layers are important parameters that control the saturation degree of the material on the slope, the rest affect the slope stability. 

In terms of distance to roads and faults, it has generally been observed that the probability of landslide occurrence increases as the distance decreases. These two layers are considered because of their correspondence to worsening of slope equilibrium conditions and contribution to terrain permeability, respectively. For all these layers, the high frequency of landslide events is localized around lower classes (i.e., class 1 and 2) which stand for lower values of distance. All these layers were obtained from the Land Information System Tasmania (LIST) except the latter one (i.e., the faults layer), which was obtained from MRT. The Euclidean distance for each layer was then re-classified into nine classes, apart from for the distance from coastal lines that was classified into 10 classes.

The normalized difference vegetation index (NDVI) and rainfall (monthly average rainfall), both from Australian Bureau of Meteorology (BOM) were other remaining continuous landslide-conditioning factors that were sub-grouped into nine and eight classes respectively due to methodology requirement. The maximum number of landslide events was recorded within class 7 of NDVI and class 3 of rainfall (the maximum rainfall is at class 8). The NDVI represents vegetation density from Jun 2017–Jun 2018 while the rainfall data is a monthly average of a 30 years rainfall. Many researchers reported that NDVI is conversely related to landslide probability [49]. However, rainfall is usually a triggering factor of landslide [50].

Finally, geology and land-use maps obtained from MRT and the Australian Land-Use and Management (ALUM) were the last groups of spatial data in the present research. Landslides are greatly controlled by the geological properties of the land surface [46]. Many researchers also emphasized the importance of land-use and land-use characteristics on the stability of slopes, and they used these parameters to assess the conditioning factors of landslides [51]. These two landslide conditioning factors were originally discrete variables. However, as the number of classes was unnecessarily high, we re-classified them into 10 classes. This gave us a more consistent dataset. In terms of geology map, it contains the detailed corresponding units (e.g., soils and rock), ages, weathering, structure, and geology source information. Most of the landslides in this area are within basalt or basalt-derived soils (class 10). In terms of land-use, landslides are observed in multiple classes of land-use considering their proximity to coastal lines.

### 3.2. Description of DoTRules with Modifications for LSM

DoTRules is a simplified rule-based algorithm for exploring rules and their corresponding uncertainties that can be applied to predict the future behavior of spatial phenomena such as in landslide susceptibility maps. Here, multiple sets of rules were implemented based on involved categorical/discrete data. The rule implementation is accomplished using a concatenation of discrete predictor variables and then measuring their relevant entropy as an estimate of reliability/uncertainty [31,52]. The procedure of DoTRules for LSM was implemented in R [53] and consisted of the following six steps.

STEP 1: Preparing test and train data.

The training set is characterized by a set of pixels *I = { i_1_, i_2_, …, i_m_}*, where each pixel *i* in the training set *I* has a value *x_ij_* for each of the independent *landslide-conditioning criteria J = {j_1_, j_2_, …, j_n_}.* In this study, 11 conditioning criteria (Table 1) exist and all of them are discretized variables. These discretized landslide-conditioning variables can be either derived from native categorical data (e.g., land-use class, geology etc.) or re-classified continuous data (e.g., distance from roads, distance to coastal lines etc.). Therefore, each *x_ij_* can adopt one of a fixed set of possible classes *H* that is specific to that criterion (Table 1). Although each criterion *j* has a different set of classes *H*, for sake of simplicity, here we do not index *H* by *j*. Each pixel *i* has also a corresponding landslide label *l_i_* from *L*{1: Landslide, 0: Non-landslide}.

STEP 2: Prioritising landslide-conditioning criteria based on Shannon entropy.

We estimated the frequency of pixels *i* within each criterion class *h* in *H* occurring within each landslide class *l* in *L*, represented as $p_{hj}^{l}$:

(1) $$p_{hj}^{l}=\frac{\sum_{i\in I}\left[x_{ij}=h][l_{i}=l\right]}{\sum_{i\in I}\left[x_{ij}=h\right]}\;\mathrm{for}\;\forall \;l\;\mathrm{in}\;L,\;h\;\mathrm{in}\;H,\;\mathrm{and}\;j\;\mathrm{in}\;J$$

where […] are Iverson brackets where [*Q*] (quantity) equals 1 if true, and 0 if false. The term $\sum_{i\in I}\left[x_{ij}=h\right]$ is the number of pixels in criterion class *h*. The $p_{hj}^{l}$ must be calculated each conditioning criterion *j* in *J*, in a similar fashion.

In this study, the Shannon entropy is the measure of uncertainty. Entropy is described as the quantitative measure of system disorder, instability, and uncertainty [54,55]. Here, we calculate the entropy of landslide/non-landslide occurrences within each landslide-conditioning criteria class *h* across all criteria *j*.

(2) $$e_{hj}=-\sum_{l\in L}p_{hj}^{l}\ln p_{hj}^{l}$$

Afterward, to calculate the entropy for each *j* in *J*, the weighted average entropy of all *h* in *H* classes (*e_hj_*) is calculated by the proportion of cells in each class:

(3) $$e_{j}=\sum_{h\in H}e_{hj}\sum_{i\in I}\left[x_{ij}=h\right]/\left|I\right|$$

where |I| is the set of pixels in the training dataset as explained in STEP 1. The landslide-conditioning factors were then ranked and prioritized according to their average entropy *e_j_*. Here, the lower entropy stands for the higher priority of landslide-conditioning factor being assessed, which is represented by the ordered set of criteria priority *J’*.

STEP 3: Creating a rule-set.

We now concatenate pixel criteria values *x_ij_* as per criteria priority *J’* in order to form a rule-set *D*. The concatenation of two or more characters is the string formed by them in a series (i.e., the concatenation of 31, A7, and 5# is 31A75#). Equation 4 illustrates the pixel values for criteria ranked in order of priority (i.e., the lowest entropy) concatenated for each pixel (row) *i*, thereby creating a unique rule for each pixel in the training dataset.

(4) $$D=\left(\begin{array}{c} x_{11}\\ x_{21}\\ \begin{array}{c} \vdots \\ x_{i1} \end{array}\\ x_{\left|I\right|1} \end{array}\right)\left|\right|\left(\begin{array}{c} x_{12}\\ x_{22}\\ \begin{array}{c} \vdots \\ x_{i2} \end{array}\\ x_{\left|I\right|2} \end{array}\right)\left|\right|\begin{array}{c} \cdots \\ \cdots \\ \cdots \\ \begin{array}{l} \cdots \\ \cdots \end{array} \end{array}\left|\right|\left(\begin{array}{c} x_{1j^{\prime }}\\ \begin{array}{c} x_{2j^{\prime }}\\ \vdots \end{array}\\ x_{ij^{\prime }}\\ x_{\left|I\right|j^{\prime }} \end{array}\right)\left|\right|\left(\begin{array}{c} x_{1\left|J^{\prime }\right|}\\ \begin{array}{c} x_{2\left|J^{\prime }\right|}\\ \vdots \end{array}\\ x_{i\left|J^{\prime }\right|}\\ x_{\left|I\right|\left|J^{\prime }\right|} \end{array}\right)=\left[\begin{array}{c} x_{11}\\ x_{21}\\ \begin{array}{c} \vdots \\ x_{i1} \end{array}\\ x_{\left|I\right|1} \end{array}\begin{array}{c} x_{12}\\ x_{22}\\ \begin{array}{c} \vdots \\ x_{i2} \end{array}\\ x_{\left|I\right|2} \end{array}\begin{array}{c} \cdots \\ \cdots \\ \begin{array}{l} \cdots \\ \cdots \end{array} \end{array}\begin{array}{c} x_{1j^{\prime }}\\ \begin{array}{c} x_{2j^{\prime }}\\ \vdots \end{array}\\ x_{ij^{\prime }}\\ x_{|I|j^{\prime }} \end{array}\begin{array}{c} x_{1\left|J^{\prime }\right|}\\ \begin{array}{c} x_{2\left|J^{\prime }\right|}\\ \vdots \end{array}\\ x_{i\left|J^{\prime }\right|}\\ x_{\left|I\right|\left|J^{\prime }\right|} \end{array}\right]=\left[\begin{array}{c} d_{1}\\ d_{2}\\ \begin{array}{c} \vdots \\ d_{i} \end{array}\\ d_{\left|I\right|} \end{array}\right]$$

Afterward, every rule within the dictionary has maintained its single landslide class $l_{i}\in L$, following the concatenation and extraction of rules. Those duplicated rules where pixels have exactly the same values for all criteria were then aggregate, leaving a parsimonious new rule-set of unique rules *D’* derived by aggregating *D*. The frequency of occurrence of all potential classes *l* in *L* was then calculated for each unique rule *d’* in *D’*:

(5) $$\left[\begin{array}{c} L_{1}\\ L_{2}\\ \vdots \\ L_{\left|D^{\prime }\right|} \end{array}\right]\begin{array}{c} \to \\ \to \\ \vdots \\ \to \end{array}\left[\begin{array}{c} f(l_{1,1})\\ f(l_{2,1})\\ \vdots \\ f(l_{\left|D^{\prime }\right|1}) \end{array}\begin{array}{c} f(l_{1,0})\\ f(l_{1,0})\\ \vdots \\ f(l_{\left|D^{\prime }\right|0}) \end{array}\right]$$

The landslide class (i.e., “0”, “1” from set *L*) with the highest frequency (i.e., the mode) is then assigned to each corresponding unique rule *d’*.

STEP 4: Calculating and mapping the uncertainty of LSM.

Considering every unique rule *d’* from our rule-set *D’*, a Shannon entropy value was then calculated based on the frequencies of each possible landslide/non-landslide class (Equation (5)), for a considered pixel, using Equation (2). This can inform both the spatial distribution of uncertainty in susceptibility mapping and provides transparent transition rules for developing better hazard mitigation plans. The spatial distribution of uncertainty was quantified and may be mapped by the entropy of each unique rule back to the pixels corresponding to each rule. Each pixel was then allocated to the landslide/non-landslide class with the highest frequency for its corresponding rule. 

STEP 5: Classifying susceptibility values of the test dataset.

Using a simplified rule-based algorithm and frequency analysis, we describe the process of generating the dictionary of trusted rules, where every single rule corresponds with its most likely class (i.e., “1” for landslide and “0” for non-landslide). The landslide occurrence class may now be predicted for the rest of the study area (i.e., the test dataset). For this purpose, we simply followed an identical process to generate required rule-sets for the test dataset, similar to the training dataset. Afterward, we matched every rule from the test data with its alike rule in the dictionary of trusted rules that are tagged with the most likely outcome (i.e., landslide/non-landslide). 

STEP 6: Handling null values

As there is always a chance to encounter new cells in the test dataset where the current combinations of criteria states are not experienced in the training set, it is possible to encounter ‘null’ values using the proposed LSM method. To resolve this issue, we sequentially removed the least important/informative (i.e., highest entropy and lowest ranked) landslide-conditioning factor or *criteria J’* from our analysis and then we re-executed steps 3–5. In this research, our proposed rule-based method was executed ten times for eleven landslide-conditioning factors of Table 1 and therefore, there were 10 rule-sets involved in susceptibility mapping of Northwest Tasmania. This way, we generated many rule-sets (i.e., a sub-rule set), which they contain fewer criteria classes in each run. The means that by moving from rule-set one (primary rule-set) to rule-set 10 (the most simplified rule-set), rule-sets will contain fewer unique rules *d’* (in the corresponding *D’*) and respectively ‘null’ records. For each pixel, existing votes of all potential classes *l* in *L* are summed and translated as landslide/non-landslide occurrence probabilities. 

STEP 7: Susceptibility classes

**Figure 3:**
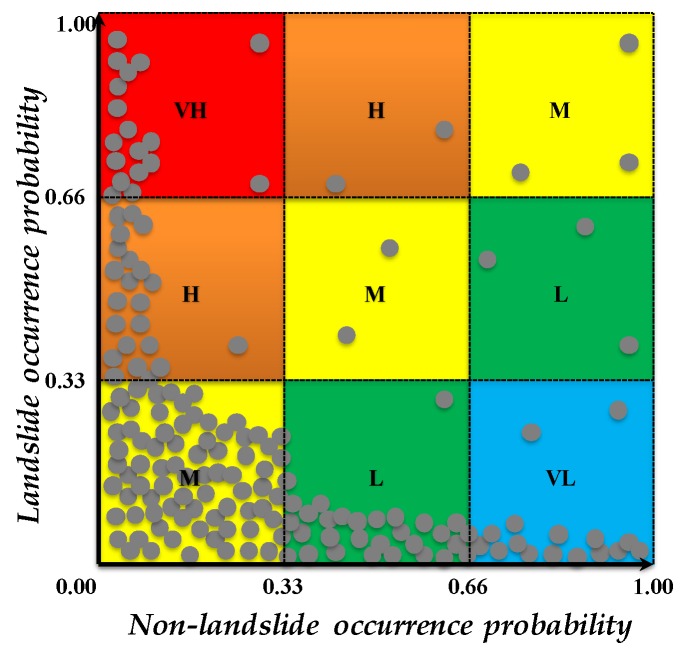
The proposed If-Then rules based on a 2D scatter plot to classify the outcomes of landslide and non-landslide occurrence probability (resistance). Here, VH, H, M, L, and VL stand for very high, high, moderated, low, and very low susceptibilities. Grey points represent sample pixels of LSM for demonstration.

Finally, to reduce the level of subjectivity in forming LSM, a set of If-Then fuzzy rules were applied for conversion of susceptibility values to susceptibility classes through merging two probability maps that are achieved by rules aimed to quantify “1” (i.e., landslide) and “0” (non-landslide) labels (Figure 3). 

As explained above, following our proposed rule-based method, every output pixel has two distinguished susceptibility “*values*” including landslide and non-landslide occurrence (resistance). Accordingly, the location of each pixel in the above 2D scatter plot determines the susceptibility “*class*” of that pixel. For instance, if the *y* (i.e., landslide occurrence probability) value of the scatter plot is above 0.66 while the *x* (i.e., non-landslide occurrence probability) value of the scatterplot is below 0.33, then the susceptibility score for that pixel is VH. Obviously, data breaks (currently 0.33 and 0.66) can be modified by users; however, the current breaks are selected to preserve equal intervals on *x* and *y* axis. 

### 3.3. Methodology Implementation

**Figure 4:**
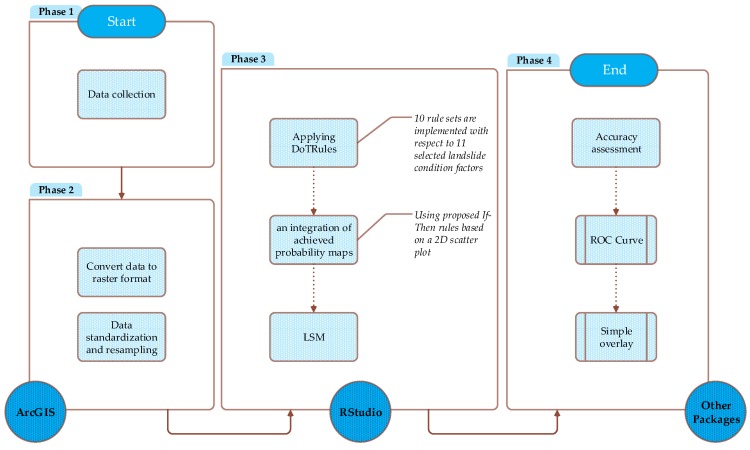
Schematic representation of the four-step methodology implementation.

Our research methodology is implemented using the following four-phase experimental design (see Figure 4). In phase 1, we collected the required data for simulation from different resources. Considering the fact that landslide-conditioning factors from Table 1 are measured not only in different units (e.g., nominal, ordinal, etc.) but also in different scales (e.g., interval, and ratio scales, etc.) [56], there was an urgent need for data resampling and standardization. This arises from the innate need to combine all landslide criteria into the single output in the evaluation process. Therefore, in phase 2, we converted our dataset into raster format with the same spatial dimensions and then we re-classified each landslide-conditioning factor to *H* discrete classes, as shown in Table 1. Referring to the existing susceptibility mapping literature, there is no optimal method for choosing the most appropriate number of classes (i.e., H) for data discretization; these are generally selected according to the preferences of the DMs [11,57]. In phase 3, the proposed method is applied for susceptibility mapping, followed by an integration of achieved probability maps using proposed If-Then rules based on the proposed 2D scatter plot (Figure 3). Finally, the achieved results were mapped and the corresponding accuracy of our LSM was assessed in phase 4. 

## 4. Results

**Figure 5:**
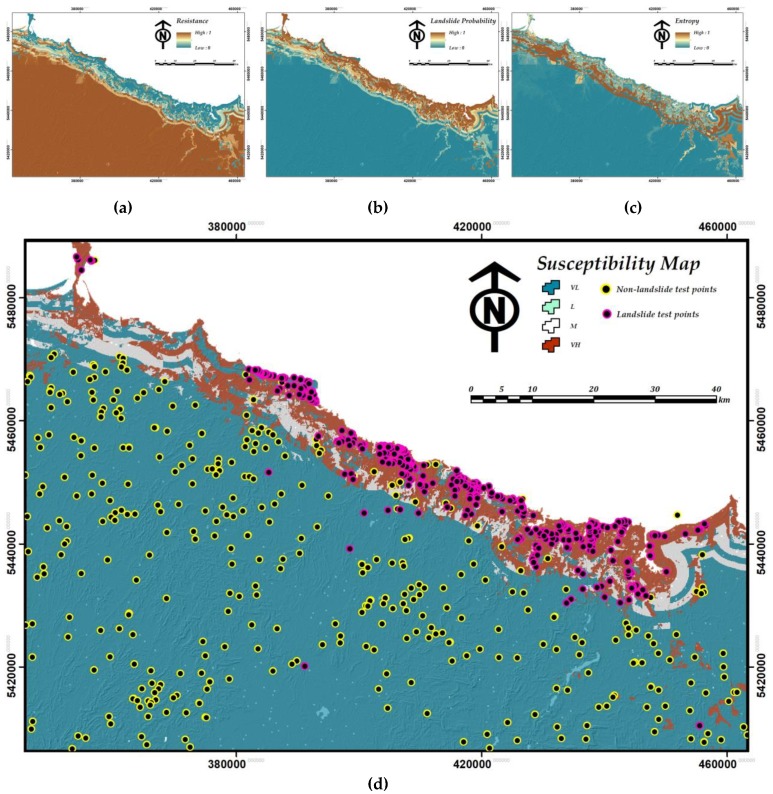
Achieved results of methodology implementation including (**a**) non-landslide occurrence probability (resistance), (**b**) landslide probability, (**c**) entropy (i.e., uncertainty) of susceptibility mapping, and (**d**) LSM.

After layer standardization of landslide-conditioning factors, the LSM was implemented and the resultant susceptibility map was produced using non-landslide occurrence probability (i.e., resistance) (Figure 5a) and landslide probability map (Figure 5b) calculated by applying modified DoTRules for LSM. Notably, as the output LSM only contains four classes of VH, M, L, VL, there are no H susceptibility classes (please see Figure 5) recognized by the defined If-Then rules. In addition, an entropy map was developed which demonstrates the uncertainty of achieved susceptibility map at a local scale (see Figure 5c). 

In terms of landslide-conditioning factors, typically, in DoTRules, the greater value of the entropy for a spatial attribute corresponds to the smaller attribute’s importance. Thus, there will be a lower discriminant power of that attribute in LSM process with higher entropy (see Table 2) [58]. Therefore, the distance from coastal lines and elevation criteria were considered as the two first important landslide-conditioning factors with lowers entropy values. As a result, the objectively obtained variables priority for LSM using Shannon entropy looks worthwhile to provide further insight into local landsliding mechanisms. 

In addition, for developing better hazard mitigation plans, susceptibility rules that are shaped within various rule-sets can be individually explored for each pixel (Table 3) and the resultant susceptibility values can be visualized (see Figure 5). Table 3 demonstrates the different variation of a distinct landslide rule in the study area that is reshaped in every iterative implementation of rule-sets for Northwest Tasmania. This is just an example of many rules that can be extracted using DoTRules, where the priority of landslide casual criteria and their discrete state/value is identified in shaping landsliding mechanisms is revealed for each corresponding pixel. This sort of detailed information is highly valuable for the development of landslide hazard mitigation plans. 

### 4.1. Validation of the Susceptibility Map Using AUC Estimate

To develop a reliable LSM procedure, it is required to validate the resultant susceptibility maps and determine their prediction capability. This is often assessed by using independent test data, which were not used through early LSM process [11]. Thus, as explained in Section 3.1, the landslide inventory database was divided into two parts, including 250 training and 391 test landslide points. Therefore, the accuracy of the proposed LSM in the study area was evaluated by calculating the receiver operating characteristics area under the curve (AUC) [11,59,60], where the frequency of known test landslide occurrence in various susceptibility classes is assessed. Here, the achieved AUC value (ranging from 0.5–1.0), is a quantitative measure of prediction accuracy, where closer values to 1 indicate that a more reliable result is achieved. On the other hand, closer values to 0.5 indicate to the less reliable result as opposed to the former state [11,61].

**Figure 6:**
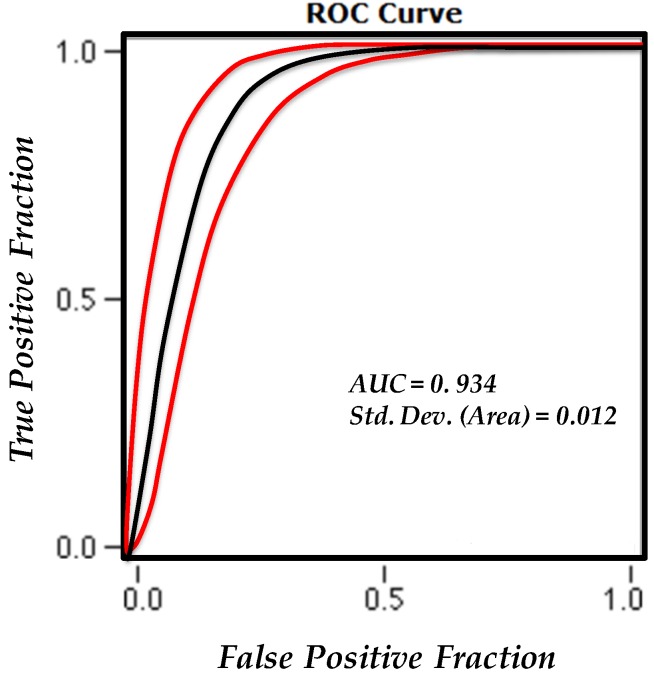
The receiver operating characteristics area under the curve for the proposed LSM.

In pursuance of further implementation of the ROC evaluation technique, a precise and comprehensive test dataset was prepared using 391 landslides and 391 randomly selected non-landslide points of the study area. Subsequently, the AUC value of 0.934 was obtained with an estimated standard error of 0.012 (see Figure 6). A further detailed summary statistics of ROC analysis is demonstrated in Table 4.

### 4.2. Validation of the LSM by Overlaying Technique

**Figure 7:**
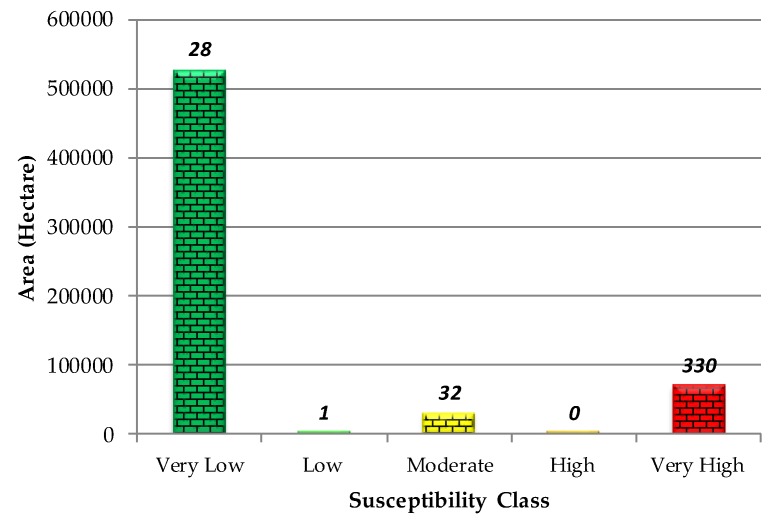
Histogram landslide susceptibility occurrence within each susceptibility class.

In the second validation process, the LSM result was evaluated using the test landslide locations, accordingly, these 391 points were overlaid on the susceptibility map of proposed hybrid GIS-based LSM (see Figure 5c). The result shows that approximately about 84.4 percent of the recorded landslides (330 landslides) occurred in the very-high susceptibility zone, which only covers less than 11 percent of the study area, while there are 29 recorded landslides in the low and very-low susceptibility zones. The number of occurred landslide events in moderate susceptibility zone is 32. In terms of the produced LSM, no pixel was assigned to the high susceptibility zone while only a few pixels were assigned to the low susceptibility zone (see Figure 7). The number of assigned pixels to each susceptibility class is subjected to change following the modification of If-Then rules within the 2D scatter plot. However, the equally-spaced low, medium and high bounds of *x* and *y* axis in Figure 3 showed the most straightforward approach.

## 5. Discussion 

Although to date statistical methodologies for implementation of LSMs are usually accurate, the existing obligation for model transparency, especially at the pixel level, is often overlooked. Nonetheless, the proper understanding of the properties of conditioning factors and scientific methodologies is also crucial to our understanding of mechanisms of landslides at local (i.e., pixel) level [15,16]. For instance, what are the priorities and class values of each conditioning factors for a considered pixel (see Table 2 and Table 3). Another disadvantage that limits the majority of LSM is the subjective re-classification of susceptibility scores into susceptibility classes (i.e., high, medium, and low susceptibility). This increases the subjectivity of LSM where elevated levels of subjectivity can affect the quality of hazard mitigation plans. Therefore, in this study, a rule-based GIS-based susceptibility mapping approach was proposed with an especial focus on model transparency at local scale while reducing the existing subjectivity of final re-classification of the developed LSM. While the transparency of the model is aimed at increasing the awareness of hazards, risk, and vulnerabilities, the proposed re-classification scheme reduce the level of subjectivity that exists in re-classification of susceptibility scores into susceptibility classes. 

### 5.1. Model Transparency and Spatial Information Extraction

Considering the high frequency of slope failure being in place in several areas of Northwest Tasmania, there was a demand to conduct an accurate landslide susceptibility map. In terms of landslides, the expected quality of mitigation plans depends not only on the model accuracy but also on model transparency and the level of information gain at a local scale [62]. DoTRules is a rule-based algorithm where rules reveal the discrete state/value of prioritized landslide-conditioning factors at a local scale (i.e., pixel-level). Although every rule can be matched to more than one pixel, as explained before, every pixel is corresponding to one and only one rule from a certain rule-set (i.e., 10 rule-sets in the current study). Each integer value composing a rule stands for criterion class *h* in *H* of a desire landslide-conditioning factor. The order of landslide-conditioning factors in each rule within every developed rule-set can be seen in Table 2, while Table 3 demonstrate examples of the same landslide rule within different rule-sets. Thus, regardless of data scale and accuracy, the present study aimed to explore local mechanisms of landslide susceptibility in Northwest Tasmania. This is an integrated strategic LSM framework with an emphasis on structuring the decision-making process problem. Within this approach, Shannon entropy was employed to determine the criteria prioritization and quantify the reliability of susceptibility rules that are applied to a pixel through the spatial domain. 

In this respect, the lower the landslide entropy of a criterion, the higher the weight is. In other words, the lower landslide entropy within certain criteria (i.e., starting from the distance to coastal lines to aspect) indicates the presence of predictive spatial frequency and vice versa. According to the obtained results, “distance from coastal lines” is the most important spatial factor in shaping landslide mechanisms throughout Northwest Tasmania (Table 2). On the other hand, “distance from mainstreams” is the least important spatial criteria considering the obtained entropy value. This only applies to the current LSM using the existing dataset. Usually, changes in the quality of landslide-conditioning factors (i.e., data resolution) may affect the computed priority order. For instance, different scales of geology maps do not belong to the same priority importance value, where a higher resolution one is usually more helpful. Similarly, the priority order of NDVI data may also be subject to change if an ideal time-series is applied. In addition, as well as global prioritization of landslide-conditioning factors using Shannon entropy, various rules that are developed within multiple rule-sets define the importance of each criterion class *h* in *H*. For example, a majority of landslide events have coincided within the first class of “distance from coastal line” criterion class. This further proves that the proposed susceptibility mapping technique is a promising tool for integrating multiple raster-based criteria for LSM, while there is not sufficient knowledge about the criteria weights with respect to landslide mechanism of the study region. The calculated entropy map as a measure of uncertainty also clarifies the reliability of susceptibility rules that are applied to each pixel. Therefore, the location of unreliable predictions that is defined with a high level of uncertainty (i.e., entropy) can be identified. This is of prominent importance in developing reliable hazard mitigation plans, where further insight into LSM procedure is provided.

### 5.2. Reducing Subjectivity of Final LSM

Regardless of the susceptibility mapping method, the re-classification of susceptibility scores into a defined susceptibility class is repeatedly applied in LSM [12,63]. This may be done by dividing the existing minimum and maximum cell susceptibility values into four or five susceptibility classes. Various approaches such as quantile [24], equal interval [13], natural breaks [11,25] etc. may be applied to delineate several susceptibility zones namely, very-high, high, moderate, low, and very-low. Although the characteristic of adopted re-classification strategy directly affects the quality of LSM, the explanation of the basis or the rationale behind the applied approach is often overlooked. Noticeably, the level of subjectivity during this procedure may harm the quality of LSM and subsequent hazard mitigation plans where the definition of very high, high, moderate, low, and very low susceptibility classes directly depends on the minimum and maximum susceptibility values and the selected re-classification approach.

Here, in this research, we proposed a set of If-Then rules based on a 2D scatter plot to interactively classify the outcome of landslide and non-landslide occurrence probability (resistance) using the proposed LSM method. As opposed to the existing approaches for re-classifying susceptibility values, in the proposed scheme, availability of the minimum number of pixels for every susceptibility class is not guaranteed. For instance, in terms of the current LSM, the “moderate” susceptibility class is 18,443 hectares, which could be even less if a smaller number of pixels meet the requirement according to Figure 3. This indicates the fact that the objective definition of different susceptibility classes is data-driven, and not susceptible to intrinsic errors of experts’ knowledge. 

### 5.3. Decision Aiding and Planning

Looking into the contribution to decision aiding, this study presents an integrated strategic susceptibility mapping procedure using an objective method which determines criteria priorities by solving mathematical models. This is executed without any consideration of the decision maker’s preferences, as it is a convention in subjective methods, such as the analytical hierarchy process (AHP) method [12], ordered weighted average (OWA) method [64], Delphi method [65] etc. In other words, this article introduces a transparent and yet objective approach that applies a rule-based approach, which could be a useful geospatial tool for integrating multiple features/attributes that affect the LSM process. This can largely compensate for the absence of expert DMs or the lack of local knowledge about the study area when it comes to producing quality LSM. In addition, a worthwhile set of information can be achieved through the implementation of DoTRules for LSM. For instance, in terms of Northwest Tasmania LSM, the strength of using “slope” as an indicator of landslide susceptibility is questioned. No doubt that slope angle plays a crucial rule in forming slope failure [39]; however, not every steep slope is susceptible to landslides. The extracted rules out of methodology implementation look beneficial to shed lights on predisposing conditions of slope failure. In case the correspondence of slope and occurred landslide events is not easy to measure, to communicate, then that it is a crude indicator and does not accommodate the significant local conditioning factors that will contribute to landslide susceptibility (e.g., distance to coastal lines, elevation). The use of low-quality slope may over-predict areas that are not truly susceptible to landslides. The same applies to other landslide-conditioning factors and often ignored prior to the implementation of LSM through a subjective approach.

Geology is also a substantial conditioning-factor for landslide susceptibility [66,67]. The underlying geology typically controls what surficial material may be available and the degree to which the substrate is susceptible to movement. However, its use as a broad indicator of landslide susceptibility throughout the Tasmanian boundaries may be significantly diluted by the current scale and accuracy. In addition, the intent of much of the available geological mapping is not focused to be utilized in LSM. In simple words, the existing geology maps of Tasmania were mainly produced for mineral exploration purposes (with an especial focus on subsurface geology). Although these maps are informative, yet, they are not always the best for sub-regional modeling of slope failure. In fact, the surface geology is often of much greater importance to landslide susceptibility; nonetheless, the existing geology map was assessed among top five conditioning factors and yet beneficial for modeling landslide susceptibility of the study area. In this study, the implementation of DoTRules for LSM considerably revealed the actual measure of importance for every landslide-conditioning factor through a data-driven approach.

### 5.4. Limitation of the Proposed Methodology in LSM

While all sort of methods based on information theory, such as our proposed algorithm, have shown considerable merits, they also have their own constraints. Although our methodology, as an objective scheme, is not dependent on experts’ knowledge and decision-making experience, it still relies on the quantification of defined landslide-conditioning factors using step-by-step mathematical computation. This is dependent on the existence of a concise and representative database. In terms of the current study, the availability of a comprehensive landslide inventory was quite advantageous in obtaining the desired outcome. However, the inefficiency of a small sample size for training purposes will be a major hindrance and particularly striking. As this problem is of great importance, therefore, exploring the ways to reduce the mentioned problem may be a fertile ground to be addressed in future studies. For instance, applying landslide polygons instead of landslide points may provide a considerable increase in the number of training pixels that may improve the quality of final LSM. We believe that more interests from researchers to apply the proposed methodology on a larger sample size of data is vital to unwrap the potential capabilities of DoTRules aimed to incorporate generalizable results in LSM.

## 6. Conclusions

In this research, we strived to present details of a novel rule-based approach for landslide susceptibility mapping (LSM) coupled with a case study of Northwest Tasmania. As long as data preparation requirements are fulfilled, we expect that our LSM methodology is also compatible with other landslide inventories that are customized for different study areas depending on the existing driving forces of slope failure. The proposed rule-based methodology showed promising results for susceptibility mapping as it tackles two major limitations. Firstly, it highlights the conditioning factors of landslide mechanism at a local scale (i.e., at pixel-level) that is often neglected in many landslide susceptibility studies. Secondly, intrinsic bias and probable errors of experts’ preferences corresponding to the subjective re-classification of LSM is minimized. Noticeably, apart from the highlighted subjectivity issue of identifying the relative importance of landslide conditioning factors, that is well regarded in the LSM literature, the subjective re-classification of susceptibility values is another disadvantage. The latter issue limits the application of LSM, and aside from heuristic approaches, also applies “statistical methods” as a downgrading factor. This is usually because of the fact that there is a risk that experts/modelers expectations affect the final status of LSM, which are far from reality. 

The projected LSM approach involves a thoughtful elaborative of landslide-conditioning factors while seeking expert opinion (i.e., for weighting or re-classification procedures) is not necessary. This is performed by constructing a mathematical approach with a high level of objectivity and promising accuracy. Considering the fact that the proposed rule-based method has the advantage of a more objective implementation, it can be used for the implementation of landslide susceptibility maps within different geographic locations where there is not enough knowledge of existing landslide mechanisms. Even if there were enough knowledge of the study area, that is always possible to face conflicting experts’ ideas or notions that are far from reality. This is a decent opportunity to contribute to decision aiding while doing LSM missions. Finally, as the application of susceptibility mapping is not only limited to landslide hazard mitigation, applying similar framework can be taken into account for the implementation of other types of susceptibility maps such as flood susceptibility maps and more.

## Figures and Tables

**Table 1:** Selected landslide-conditioning factors based on literature review, relevant data source, description and number of discrete classes (H).

Criteria	Data Source	Description	H
**1. Slope**	Mineral Resources Tasmania (MRT)	This is the slope angle derived from a digital elevation model (DEM) of the 10 metre Lidar DEM.	9
**2. Aspect**	Mineral Resources Tasmania (MRT)	The compass direction that a slope faces derived from the same source as slope.	9
**3. Mainstreams**	Land Information System Tasmania (LIST)	The relative Euclidian distance of each desired pixel from the closet mainstream.	9
**4. Coastal lines**	Land Information System Tasmania (LIST)	The relative Euclidian distance of each desired pixel from coastal lines.	10
**5. NDVI**	Australian Bureau of Meteorology	The normalized difference vegetation index (NDVI) representing vegetation density and condition from Jun 2017 to Jun 2018.	9
**6. Rainfall**	Australian Bureau of Meteorology	A monthly average of a 30 years rainfall (base climatological datasets) from 1961–1990.	8
**7. Road**	Land Information System Tasmania (LIST)	The relative Euclidian distance of each desired pixel from the closet road.	9
**8. Geology**	Mineral Resources Tasmania (MRT)	This Tasmania Geology map is derived from the 1:250,000 scale digital geology of Tasmania.	10
**9. Faults**	Mineral Resources Tasmania (MRT)	The relative Euclidian distance of each desired pixel from the closet geological fault.	9
**10. Elevation**	Mineral Resources Tasmania (MRT)	The representation of the land surface elevation from 10 metre Lidar source.	9
**11. Land use**	the Australian Land Use and Management (ALUM)	The Tasmanian land use map containing 116 land-use sub-classes for the current study area.	10
**12. Landslides**	Mineral Resources Tasmania (MRT)	A number of 641 records containing both active and inactive landslides.	-

**Table 2:** Landslide conditioning variables priority rank and the corresponding entropy values.

Rank	Variable Name	Entropy Score
**1**	Coastal lines	0.272
**2**	Elevation	0.378
**3**	Rainfall	0.437
**4**	Land use	0.488
**5**	Geology	0.540
**6**	NDVI	0.557
**7**	Road	0.579
**8**	Slope	0.659
**9**	Faults	0.659
**10**	Aspect	0.664
**11**	Mainstreams	0.671

**Table 3:** Accuracy metrics of implemented LSMs for Northwest Tasmania.

Rule-set ID	Rule (Composed of Discrete H Values)	Frequency	Matching Landslides	Entropy
1	1_1	130	125	0.163
2	1_1_3	79	75	0.200
3	1_1_3_9	35	34	0.129
4	1_1_3_9_10	25	25	0
5	1_1_3_9_10_7	10	10	0
6	1_1_3_9_10_7_1	7	7	0
7	1_1_3_9_10_7_1_2	1	1	0
8	1_1_3_9_10_7_1_2_9	1	1	0
9	1_1_3_9_10_7_1_2_9_5	1	1	0
10	1_1_3_9_10_7_1_2_9_5_2	1	1	0

**Table 4:** Accuracy metrics of implemented LSMs for Northwest Tasmania.

Summary Statistics	Achieved Values
Number of Cases	782
Number Correct	677 (86.5% of total)
AUC	0.934
Std. Dev. (Area)	0.012
Accuracy	86.6%
Sensitivity	92.6%
Specificity	80.6%
Pos Cases Missed	29
Neg Cases Missed	76
